# Nutrient Intake in Children 4–13 Years Old in Ibadan, Nigeria

**DOI:** 10.3390/nu13061741

**Published:** 2021-05-21

**Authors:** Marie Tassy, Alison L. Eldridge, Rasaki A. Sanusi, Oluwaseun Ariyo, AnuOluwapo Ogundero, Tolu E. Eyinla, Dantong Wang

**Affiliations:** 1 Nestlé Institute of Health Sciences, Nestlé Research, Vers-chez-les-Blanc, 1000 Lausanne, Switzerland; Marie.Tassy@rd.nestle.com (M.T.); Alison.Eldridge@rdls.nestle.com (A.L.E.); 2 Department of Human Nutrition and Dietetics, University of Ibadan, 200284 Ibadan, Nigeria; sanusiadegoke2003@gmail.com (R.A.S.); ariyoseun@gmail.com (O.A.); anuoluwaogundero@gmail.com (A.O.); toluemma@ymail.com (T.E.E.)

**Keywords:** nutrient intake, Nigeria, school age children, inadequacy

## Abstract

The paucity of adequate data on dietary and nutrient intakes of school-age children is a barrier to addressing malnutrition and associated risks in Nigeria. This study included 955 children aged 4–13 years from Ibadan, Nigeria, using a stratified random sampling design. Information on family socio-demographic characteristics was reported, and child anthropometrics were measured. Dietary intake data were collected using a multi-pass 24 h dietary recall method; 20% of subjects completed a second 24 h recall to estimate usual nutrient intakes. Means and distributions of usual intakes of energy and nutrients as well as prevalence of inadequacy were estimated. Usual energy intake (kcal/day) was 1345 and 1590 for younger (4–8 years) and older (9–13 years) age groups, respectively. The macronutrient intakes of most children did not conform to Adequate Macronutrient Distribution Ranges (AMDRs), which were characterized by a higher proportion of energy from carbohydrates and lower proportion from total fats. Protein intake was largely within the AMDR. Compared to recommendations, over 60% of 4–8-year-old children had inadequate intakes of calcium, copper, iron, folate, and vitamins A, D, and E. There were more micronutrient inadequacies in the older children. This study identifies nutrition gaps and suggests future research and education to improve child nutrition in Nigeria.

## 1. Introduction

Nigeria is the most populous country in Africa with over 200 million people; about 27% of the population is between 5 and 14 years of age [1]. Understanding nutrient intakes is a challenge in this age group, as the Nigeria Demographic and Health Survey (NDHS) focuses on children under the age of 5 years and women of reproductive age without including school-age children [2]. In addition, while the NDHS provides important information about key nutrition and health indicators related to acute malnutrition, vaccination coverage, malaria, diarrheal diseases, infant feeding, and maternal health, the survey does not provide detailed dietary and nutrient intake data.

Several regional studies have been conducted in Nigeria that focus on the nutritional status of school-age children [3]. These studies, from a systematic review conducted from 2005 to 2015, showed high rates of zinc (63%) and iodine deficiency (59%), and lower rates of vitamin A (16%), iron deficiency (12%), and iron deficiency anemia (14%) in Nigerian school-aged children [3]. Adequate dietary intakes are needed to help address these deficiencies, but more data on the nutrient intakes of school-age children are essential for identifying additional nutritional risks.

Previous dietary intake studies among Nigerian school children have shown inadequate intakes as well. Inadequate intakes of vitamin A, iron, iodine, calcium, and vitamin C were reported among 7–11-year-olds from Kaduna in the northern part of Nigeria [4]. In a rural community in Enugu State in Southeast Nigeria, children 13–15 years old consumed about 65% of the daily reference nutrient intakes (RNI) for iron, though younger children had more adequate intakes [5]. One-quarter of the children 6–15 years of age were below the RNI for vitamin A [6], and low intakes of calcium and niacin were also observed [7]. In a study among 10–19-year-olds from lower socio-economic schools within Ogun State, Southwest Nigeria, significant numbers of children had intakes below the RNIs for iron, vitamins B6, B12, and calcium [8]. Low consumption of iron, folate, and calcium was also reported for adolescent girls 12–18 years old from Akwa Ibom State, which is located in the coastal southern part of the country [9].

While these previous studies help to identify some nutritional gaps in different parts of the country, they provided only mean intakes of a limited number of nutrients. In addition, some of the sample sizes are quite small (e.g., less than 100 subjects per group) or limited to small geographical areas. A more comprehensive assessment in one of the most populated areas of the country is needed.

The purpose of this study was to fill the knowledge gap on nutrient intakes in a large sample of school-age children in Ibadan, Nigeria. Since previous research has shown differences in intakes of some nutrients by age [7], the current study disaggregated the nutrient intake data of school age children by age group, 4–8 years and 9–13 years old, and provided an assessment of inadequate intakes for a broad range of nutrients. Inadequacy was also evaluated across socio-economic levels to gain insight into the effect of income on dietary adequacy.

## 2. Materials and Methods

### 2.1. Study Design

The Ibadan Kids Nutrition and Health Study (I-KNHS) was descriptive, cross-sectional dietary intake study that adopted a stratified random sampling design. A total of 955 children aged 4–13 years were selected from all the five urban local government areas of Ibadan comprising 59 wards and various social strata. Information on socio-demographic characteristics of households and caregivers, child characteristics, lifestyle parameters, feeding practices, dietary patterns, and intakes were collected. Sick/ill children and children whose primary caregiver did not give assents were excluded from the study. The study was conducted according to the guidelines in the Declaration of Helsinki. All the procedures involving human subjects were approved by the University of Ibadan/University College Hospital Ethical Committee (UI/UCH EC Registration Number NHREC 105/01/2008a)-UI/EC/19/0107 (15 May 2019) and Oyo State Ministry of Health (AD 13/479/1149 (18 March 2019). Written informed consent was obtained from the primary caregiver of each child participating in the study.

All information collected was obtained through face-to-face interviews with each child and caregiver of each child in their home. A pilot study with 100 subjects was conducted to test the study procedures, evaluate the study instruments, and assess the actual data collection process. Prior to the pilot study and after the pilot, all interviewers were trained on study procedures and interviewing techniques using a standard protocol. The home visit included a general questionnaire, assessment of anthropometric characteristics of both caregiver and index child, and administration of a multi-pass 24 h dietary recall for the child. The general questionnaire provided information on family demographic and socio-economic characteristics such as child age, birth weight of the child, education and occupation of parents, and monthly household income (per capita).

One 24 h dietary recall was completed for all children, and 20% completed a second/repeat 24 h recall so that usual nutrient intakes could be estimated. Food, water, and beverage intakes and portions consumed were collected using a 24 h dietary recall questionnaire adapted for the I-KNHS study. Respondents aged 12 years and above were asked to recall what they had eaten in the previous 24 h; respondents aged 6–11 years were interviewed with the assistance of the caregiver or an adult familiar with the child’s intake, and parents/caregivers responded on behalf of children less than 6 years of age. A food instruction booklet including a food measurement guide was used to aid interviewers in obtaining detailed information on the types of food eaten and the quantity in household measures.

All data were collected using an e-questionnaire on the Open Data Kit (ODK) platform and a Tablet. Physical activity level of the children was assessed using the Physical Activity Questionnaire for Older Children (PAQ-C) and Adolescents (PAQ-A) [10]. We collected anthropometric data (body weight and height) using standard procedures and analyzed using World Health Organization (WHO) Anthro and Anthro Plus software [10]. Obesity was defined as BMI-for-age > 2 Standard Deviations (SD) above the WHO growth standard median; overweight was defined as a BMI-for-age >1 SD above the median; and thinness was defined as BMI-for-age < 2 SD below the median [11]. A child was considered as stunted when the Height-for-Age index was <2 SD below the median. Analysis of food intake was performed using ESHA’s Food Processor ^®^ Nutrition Analysis software version 11.7.1 (ESHA Research, Salem, OR, USA).

### 2.2. Food Composition Database

Energy and nutrient content of foods and beverages consumed was estimated using the West African Food Composition Table (WAFCT), updated in 2019, which contains the composition in 27 nutrients of 472 foods from the Western Africa region [12,13]. When a food product was not available in the WAFCT, the Food and Nutrient Database for Dietary Studies (FNDDS) from the US Department of Agriculture was used. Laboratory analyses were performed to establish the nutrient composition of commonly consumed food items, such as traditional soups and stews (*Efo Riro* and *Ewedu*), which were missing from both databases. We also imputed missing nutrient values using the USDA FNDDS to get more complete intake estimates for a wider range of nutrients.

### 2.3. Reference Values

For the evaluation of energy intake, the Estimated Energy Requirement (EER) was calculated for each individual using Institute of Medicine (IOM) equations [14] considering age, sex, body weight, height, and physical activity level (PAL). PAL was defined as active for all children based on the assessment of local investigators, since active physical activity is common after school in the region. Since the diet of Nigerian children is predominately plant based, the Recommended Nutrient Intakes (RNIs) of iron and zinc with low bioavailability from the WHO and FAO were used in the analysis [15]. The maximum level of sodium intake recommended by the WHO is 2 g/day sodium in adults and should be adjusted downward based on the energy requirements of children [16]. The Nigerian adult energy intakes were estimated based on data from a household survey [17], and the reference values for maximum sodium intake in children used in this study were adjusted according to the recommendation.

### 2.4. Statistical Analysis

All statistical analyses were performed using SAS^®^ Life Science Analytics Framework (version 5.2.1, SAS Institute Inc., Cary, NC, USA). Mean energy intakes and standard deviation on both recording days were calculated per age group. Children having mean energy intakes above or below three standard deviations for their age group mean were considered as outliers and removed from the analysis. A total of 946 children were included in the final analysis.

Mean and usual intake distributions of energy and nutrients as well as prevalence of inadequacy were estimated using the macros developed by the National Cancer Institute [18]. Nigeria does not have specific recommended nutrient reference values, so nutrient adequacy was assessed by comparing nutrient intakes to the dietary reference intakes established by the US IOM [19]. When available, the prevalence of inadequacy in a group was estimated as the proportion of individuals with usual intakes below the Estimated Average Requirement (EAR), using the EAR cut-point method. The average age at menarche among Nigerian girls is 13 years old [20,21], so we did not consider the iron requirements to be skewed for our population, and the cut-point method was used for iron in this study [22]. Intakes of macronutrients were evaluated as percentage of total energy intake, and inadequacy or excessive intake was classified as less than the lower limit or higher than the upper limit of the Acceptable Macronutrient Distribution Ranges (AMDR). Assessment of nutrient adequacy was also computed by age groups (4–8 years and 9–13 years of age) and socio-economic level. The socio-economic classification (SEC) was adapted from the urban classification system reported by the Market Research Society of India (MRSI), which uses education levels and occupational criteria of the respondent as a measure of socio-economic classification into seven groups (A1–E2). Then, the seven groups were summarized into three major categories: “high” which refers to SEC A and B, “mid” refers to SEC C, while “low” refers to SEC D and E [23].

Fisher’s exact test was used to compare the prevalence of nutrient inadequate intakes between 4–8 and 9–13-year-old groups. Chi-square and chi-square for trend tests were used in the comparison of prevalence of nutrient inadequate intakes among different SECs. Bonferroni correction was applied to adjust for multiple comparisons.

## 3. Results

### 3.1. Demographics

Children were divided in two age groups: 4–8 years (*n* = 510) and 9–13 years (*n* = 434) with 49% and 48% boys in each age group, respectively (Table 1). Among younger children, 25% children belong to the highest socio-economic level, 37% belong to the middle, and 38% belong to the lowest. A total of 43% of 9–13-year-olds belong to the lowest economic level and 34% belong to the middle socio-economic level. A total of 11% of 4–8-year-olds and 18% of 9–13-year-olds were stunted. In this sample, 7% of 4–8-year-olds and 5% of 9–13-year-olds were at risk for overweight or obesity.

### 3.2. Energy, Usual Nutrient Intakes, and Inadequacy for 4–8 and 9–13-Year-Old Children

**4–8-year-old children.** Energy intakes among younger children averaged 1345 kcal/day (Table 2). Macronutrient intakes of most 4–8-year-old Nigerian children do not conform to AMDRs, with 69% of children having more than 65% of energy intakes coming from carbohydrates and 96% of children with less than 25% of energy intakes coming from fats. However, protein intakes are largely within the acceptable range with only 3% of younger children consuming inadequate intakes of proteins.

A total of 99%, 62%, and 44% of children were below the EAR for calcium, iron, and zinc, respectively. Usual intakes of iron were 9.4 mg/day, whereas the EAR for iron in a low-bioavailability diet was set at 11.2 mg/day. The mean intakes of zinc was 9.3 mg/day, the same as the EAR for zinc on a low-bioavailability diet.

Almost all children were above the AIs for magnesium and manganese but only 28% exceeded the AI for fluoride. More than 90% of children had pantothenic acid, vitamin E, and vitamin D values below the EAR. The usual intake of folate was 138 μg DFE/day, and 74% of the children were below the EAR of 160 μg DFE/day. In the study, 67% of children were below the EAR for vitamin A. Intake of B vitamins was closer to recommendations for this age group, with less than 35% below the EAR. Half of the children were above the AI for biotin, and 27% were above the AI for vitamin K.

**9-13-year-old children**. Usual nutrient intakes are also low compared to recommendations among older children (Table 3). The mean energy intake for 9–13-year-olds was 1590 kcal/day. Carbohydrates intakes exceeded the AMDR for 84% of these children, whereas nearly all were below the AMDR for fats. Seven percent of older children consumed inadequate intakes of proteins.

More than 90% of 9–13-year-olds had intakes below the EAR for calcium and iron. The usual intake of calcium was 301 mg/day, which is far below the EAR of 1100 mg/day. Usual iron intakes were 11 mg/day, which is lower than the recommendation for a low bioavailability diet of 16.4 mg/day for boys and 16.8 mg/day for girls of this age group. A total of 78%, 72%, and 58% of children had intakes of magnesium, phosphorus, and zinc below EAR, respectively.

Only 2% of 9–13-year-old children had intakes above AI for potassium. Almost all children had inadequate intakes of folate, vitamin A, C, and D. Usual intakes of the latter nutrients were 153 μg DFE/day, 271 μg RAE, 31 mg/day, and 1.2 μg/day, respectively. Between 55 and 80% of children had intakes of thiamin, riboflavin, vitamin B12, and vitamin C below the EAR. Less than 30% of children had inadequate intakes of niacin and vitamin B6.

### 3.3. Inadequacy by Age and Socio-Economic Level

Nearly all children, regardless of age, were below the EAR for calcium, copper, vitamin D, and vitamin E (Table 4). For other nutrients, the percentage of inadequacy was significantly higher in the older compared to younger age groups. Socio-economic status played little role in nutrient adequacy, as no significant differences were observed across SES categories for either age category.

## 4. Discussion

This study reported nutrient intakes from a large sample of Nigerian school-age children in Ibadan, the capital city of Oyo state. A stratified random sampling design was used to identify a robust sample across different socio-economic classes in the area. Anthropometric measures were collected, and usual nutrient intakes were estimated to present a robust view of the nutritional issues facing these children. It is well known that malnutrition causes issues in child growth. In this study of Nigerian school children, stunting is still a great concern, with prevalence of 11% in 4–8-year-olds and 18% among and 9–13-year-olds. The prevalence of obesity is relatively low with prevalence of 2.2% and 1.6% in 4–8-year-olds and 9–13-year-olds, respectively. Earlier studies have shown similarly high burden of stunting and low prevalence of obesity in the various regions of Nigeria [24,25,26]. Reported energy intakes were about 17% below the EER for children in both age groups. Some amount of under-reporting is typical from 24 h recalls [27], but it is also important to note that the low energy reported for some children in this population may be related to their underweight status.

Protein intakes were generally adequate for both younger and older children in this study. We compared protein intakes with the AMDR, whereas previous studies in Nigeria compared protein intakes to the RNI [7,8] or presented average protein intakes as a percentage of total energy [28]. Amounts of protein consumed by school-age children in Ibadan were similar to those reported in previous studies in Enugu State [7] and Ogun State [8,28]. We also observed a decrease in protein intakes in older school children compared to the younger ones, which is similar to findings from the study in Enugu State [7].

Fat intakes in our study were low and largely below the AMDR for children 4–13 years old. Previous studies in Nigerian children and adolescents reported fat intakes ranging from 44.3 to 84.8 g/d [7,8,28], whereas we saw intakes at closer to 30 g/d. One possible explanation is that our sample included younger children, beginning at the age of 4 years, whereas the youngest children in the previous studies were 6 years old [7], 8 years old [28], and 10 years old [8]. However, even among older children, the fat intakes reported in our study tended to be lower than previous reports. It is possible that fats used in food preparation were under-estimated or that dietary habits in Ibadan are slightly different from those in other areas of southern Nigeria.

About 67% of the daily energy came from carbohydrates, which is higher than the upper boundary of AMDR (65%), but total sugars intake was ≤5%, which is consistent with WHO recommendations [29]. Despite low sugar intakes, fiber was reported to be about half of recommended intake levels. Our study is the first to report fiber and total sugars intake in the region.

The most recent previous studies of Nigerian school children reported mean intakes of calcium, iron, zinc, vitamin A, some B vitamins, and vitamin C [4,7,8]. These studies have reported large percentages of children below the RNI for iron, calcium, vitamin B6, B12 [8], vitamin A, and iodine [4]. However, simply comparing mean intakes to an RNI does not identify the proportion of the population at risk for inadequacy. To our knowledge, ours is the first study reporting usual nutrient intakes in the region. We were able to identify that virtually all children in both age groups were below the EAR for calcium, vitamin D, and vitamin E. Since vitamin D can also be made by exposure to sunlight, it is not clear the extent to which the low intakes put these children at risk for deficiency, but a high percentage of inadequate intakes of calcium is especially worrisome for child bone growth and development.

Previous studies showed high sodium intake in Nigeria adults [30,31]. In this study, the average sodium intake of 4–13-year-old children was 1300–1560 mg. Compared to the recommendation adjusted by energy, around 30% of children had excessive sodium intake. However, the possibility of underestimation exists due to the difficulties in collecting salt used in cooking. Despite the high sodium intake, more than 95% of the children had iodine intakes below the EAR. A similar finding has been reported among school-age children in Kaduna, Northern Nigeria, where about 92% had inadequate iodine intake [4]. The low intake of iodine is unexpected following widespread iodization of salt in the country. As of 1994, 97% of edible salt in Nigeria was iodized, and more than 95% of households consumed salt iodized to the level of 50 ppm [32]. However, follow-up studies across the various regions of the country have shown a decline over time. In a study conducted in Enugu, Southeast Nigeria, 53% of salt samples at the household level had inadequate iodine content, including 34% with zero iodine content [33]. Likewise, more than one-third of households in Cross River consumed salt with inadequate iodine concentration [3,34], and about 75% had urinary iodine that is suggestive of mild to severe iodine deficiency [34]. These observations have been attributed to the improper use of table salt in cooking and poor handling by the retailers.

In this study, we found high levels of inadequate intakes of some other micronutrients despite the fortification of staple foods in Nigeria. These locally grown foods, which are not industrially processed and therefore not fortified, constitute the bulk of the diet. The mean iron intake in our study is similar to the mean reported among children 7–11 years in Kaduna, North Western Nigeria [4] and lower than reported in among children in South Eastern Nigeria [5]. However, in all cases, mean intakes were below recommendation. Similarly, low intakes of zinc, vitamin A, calcium, vitamin B12, and B1 have been reported among school age children in Enugu and Ile-Ife, Southern Nigeria [6,7,35].

We observed that older children in our study had higher inadequacies than younger children. This is likely related to the increase in nutrient requirements for 9–13-year-olds compared to 4–8-year-olds [19]. These increased requirements mean that a very high proportion of 9–13-year-olds are also at risk for inadequate intakes of iron, folate, and vitamin A. About three-quarters of the older children in this sample also had inadequate intakes of magnesium, phosphorus, thiamin, riboflavin, and vitamin C. We also found that nutrient intakes did not differ by socio-economic class. This could indicate that similar amounts are spent on food regardless of SEC, or potentially, the method used to classify households based on education levels and occupation was insufficient to define SEC. Further study is needed to verify this aspect.

This study has several strengths. First, a robust sampling design was used to collect a representative sample of nearly 1000 children from Ibadan. Ibadan is an urban center and typically has a representative population of nearly all the ethnic nationalities in Nigeria. Although Ibadan is not representative of the entire country, it is representative of the urban centers in Nigeria, and the food consumption and nutrients intake of children in Ibadan are typical of children in other major cities in the country. Secondly, data were collected from children in their homes, rather than in schools, also increasing the generalizability of the results. Thirdly, a recently updated food composition table for the region that was enhanced with commonly consumed recipes and imputed values for missing nutrients was used. Lastly, for the first time in the region, usual nutrient intake estimations were used to assess adequacy of nutrient intakes. However, there are limitations. Children may have difficulty reporting on foods, such as fats, sugars, or salt used in food preparation, which may have underestimated intakes of these components. Parents, who reported on intakes of the younger children, may also have underestimated intakes for foods consumed outside of the home. Finally, our sample may not be representative of children living in more rural regions of the country. Nevertheless, it serves to identify important nutrient gaps in this vulnerable population.

## 5. Conclusions

Undernutrition continues to be a public health concern among Nigerian school-age children. The fact that older children are at higher risk for under-consumption of key nutrients is an important finding. Surprisingly, we did not see differences by SEC, and this merits further research. Our focus was on urban school children in Ibadan, and while our findings may be typical of children in other urban areas of the country, future research should be done to confirm this and to understand if the nutritional issues facing rural children would be similar. Our research identifies several opportunity areas for improvement. Children and families could be educated about food sources of nutrients in the diet, especially for shortfall nutrients such as iron, calcium, folate, vitamins C, D, and E, and encouraged to consume foods from all food groups. Nutrition education efforts could help families to identify and incorporate lower-cost nutrient dense foods into household meals. Schools could evaluate the possibility of lunch programs that help children meet nutritional needs during the school days or improve the nutritional quality of existing programs.

## Figures and Tables

**Table 1:** Ibadan Kids Nutrition and Health Study: sample population characteristics and estimated energy requirements.

	Total *n* = 944	4–8-Year-Olds *n* = 510		9–13-Year-Olds *n* = 434	
Characteristic	Number	Number	%	Number	%
**Gender**					
Male	458	250	49.0%	208	47.9%
Female	486	260	51.0%	226	52.1%
**Socio-Economic Level**					
Highest	228	127	24.9%	101	23.3%
Middle	334	188	36.9%	146	33.6%
Lowest	382	195	38.2%	187	43.1%
**Body Mass Index**					
Obese	18	11	2.2%	7	1.6%
Overweight	37	24	4.7%	13	3.0%
Normal weight	740	417	81.8%	323	74.4%
Thinness	149	58	11.4%	91	21.0%
**Stunted**	135	56	11.0%	79	18.2%
EER (kcal) *	-	1580		1860	

* The following Institute of Medicine (IOM) equations were used to calculate Estimated Energy Requirements (EER) [14]: Boys 3–8 years old: EER = 88.5 − (61.9 × age [year]) + 1.26 × (26.7 × weight [kg] + 903 × height [m]) + 20; Boys 9–13 years old: EER = 88.5 − (61.9 × age [year]) + 1.26 × (26.7 × weight [kg] + 903 × height [m]) + 25; Girls 3–8 years old: EER = 135.3 − (30.8 × age [year]) + 1.31 × (10.0 × weight [kg] + 934 × height [m]) + 20; Girls 9–13 years old: EER = 135.3 − (30.8 × age [year]) + 1.31 × (10.0 × weight [kg] + 934 × height [m]) + 25.

**Table 2:** Distribution of usual nutrient intakes and percentage of inadequacy in 4–8-year-old Nigerian children.

Nutrient	Mean	SD	10th Percentile	25th Percentile	50th Percentile	75th Percentile	90th Percentile	EAR/AMDR Reference Boys/Girls	AI Reference Boys/Girls	UL/AMDR Reference Boys/Girls	Percentage Above or Below EAR/AI/AMDR
Energy (kcal)	1345	197	1099	1207	1335	1473	1604				
Carbohydrates (g)	223.6	38.1	176.5	196.7	221.1	247.9	273.8				
Energy from carbs (%E)	66.8	3.9	61.8	64.2	66.9	69.5	71.8	45		65	69% > AMDR
Total sugars (g)	17.3	9.0	7.6	10.8	15.5	21.9	29.2				
Energy from total sugars (%E)	5.0	2.6	2.2	3.1	4.5	6.3	8.4				
Fibers * (g)	8.1	2.5	5.0	6.3	7.9	9.7	11.5		18		0.1% > AI
Fats (g)	28.8	6.5	20.9	24.1	28.2	32.8	37.4				
Energy from fats (%E)	18.9	3.4	14.8	16.5	18.6	21.0	23.3	25		35	96% < AMDR
Saturated fats (g)	5.4	2.5	2.6	3.6	5.0	6.7	8.7				
Energy from SFA (%E)	3.5	1.3	2.0	2.6	3.4	4.3	5.2			10	99% < AMDR
Protein (g)	43.7	6.6	35.4	39.0	43.3	48.0	52.4				
Energy from proteins (%E)	13.0	1.7	10.9	11.9	13.0	14.1	15.2	10		30	3% < AMDR
Calcium (mg)	296.7	108.8	169.1	217.8	282.8	360.8	442.1	800			99% < EAR
Copper (mg)	12.9	5.8	6.2	8.6	12.0	16.2	20.6	340			99% < EAR
Fluoride (mg)	1.5	5.2	0.0	0.1	0.3	1.2	3.5		1		28% > AI
Iodine (μg)	19.6	24.2	3.4	6.2	12.2	23.7	42.8	65			96% < EAR
Iron ** (mg)	9.4	2.2	6.6	7.8	9.2	10.7	12.3	11.2			62% < EAR
Magnesium (mg)	145.9	32.0	106.2	123.3	143.9	166.5	188.2	110			13% < EAR
Manganese (mg)	4.1	1.4	2.4	3.1	4.0	5.0	6.0		1.5		99% > AI
Phosphorus (mg)	781.1	214.5	531.4	627.2	753.6	905.3	1065.5	405			99% > AI
Potassium (mg)	1092.5	267.5	766.7	901.5	1069.8	1259.4	1447.1		2300		0% > AI
Selenium (μg)	43.8	22.3	18.4	927.4	40.2	56.3	73.8	23			17% < EAR
Sodium (mg)	1303.8	195.4	1058.5	1167.4	1295.5	1431.6	1559.6		1000	1360	95% > AI
Zinc ** (mg)	9.3	3.3	5.5	7.0	8.9	11.3	13.7	9.3			44% < EAR
Biotin (μg)	14.6	9.7	5.1	7.8	12.3	18.9	27.1		12		51% > AI
Folate (μg DFE)	137.9	39.4	91.1	109.6	133.5	161.5	190.2	160			74% < EAR
Pantothenic acid (mg)	1.7	0.6	0.9	1.2	1.6	2.1	2.5		3		96% < EAR
Vitamin A (μg RAE)	249.0	72.9	160.4	196.7	242.5	294.4	346.0	275			67% < EAR
Thiamin (mg)	0.6	0.1	0.4	0.5	0.6	0.7	0.8	0.5			28% < EAR
Vitamin B12 (μg)	1.4	0.7	0.6	0.9	1.3	1.8	2.3	1			33% < EAR
Riboflavin (mg)	0.7	0.2	0.4	0.5	0.6	0.8	1.0	0.5			25% < EAR
Niacin (mg)	10.8	3.8	6.3	8.0	10.3	13.0	15.8	6			8% < EAR
Vitamin B6 (mg)	0.9	0.3	0.6	0.7	0.9	1.1	1.3	0.5			4% < EAR
Vitamin C (mg)	29.0	5.5	22.2	25.1	28.7	32.6	36.3	22			9% < EAR
Vitamin D (μg)	1.3	1.0	0.3	0.6	1.0	1.7	2.5	10			99% < EAR
Vitamin E (mg)	4.1	0.9	3.0	3.5	4.1	4.7	5.4	6			97% < EAR
Vitamin K (μg)	46.4	16.7	27.1	34.4	44.1	56.0	68.6		55		27% > AI

Abbreviations used: Standard Deviation (SD); Estimated Average Requirement (EAR); Adequate Macronutrient Distribution Ranges (AMDR); Adequate Intake (AI); Saturated Fatty Acids (SFA); and Dietary Folate Equivalents (DFE). * Based on IOM recommendation of 14 g/1000 kcal of fibers and adjusted by the median energy of each age group; ** The recommended nutrient intakes of iron and zinc with low bioavailability from WHO and FAO.

**Table 3:** Distribution of usual nutrient intakes and percentage of inadequacy in 9–13-year-old Nigerian children.

Nutrient	Mean	SD	10th Percentile	25th Percentile	50th Percentile	75th Percentile	90th Percentile	EAR/AMDR Reference Boys/Girls	AI Reference Boys/Girls	UL/AMDR Reference Boys/Girls	Percentage Above or Below EAR/AI/AMDR
Energy (kcal)	1590	295	1221	1383	1576	1783	1977				
Carbohydrates (g)	271.1	50.0	208.6	236.0	268.6	303.7	336.6				
Energy from carbs (%E)	68.6	3.7	63.9	66.2	68.7	71.2	73.3	45		65	84% > AMDR
Total sugars (g)	17.9	0.0	17.9	17.9	17.9	17.9	17.9				
Energy from total sugars (%E)	4.5	0.5	4.0	4.2	4.5	4.8	5.1				
Fibers * (g)	10.1	2.6	6.9	8.2	9.9	11.7	13.5		24/21		0.01% > AI
Fats (g)	31.9	7.4	23.1	26.7	31.3	36.5	41.7				
Energy from fats (%E)	17.7	3.1	14.0	15.6	17.5	19.7	21.8	25		35	99% < AMDR
Saturated fats (g)	5.6	1.8	3.4	4.3	5.4	6.7	8.1				
Energy from SFA (%E)	3.2	0.9	2.1	2.6	3.1	3.7	4.4			10	99% < AMDR
Protein (g)	50.0	14.0	32.9	39.9	48.7	58.7	68.6				
Energy from proteins (%E)	12.4	1.7	10.3	11.2	12.3	13.5	14.6	10		30	7% < AMDR
Calcium (mg)	301.4	68.0	218.8	252.9	295.5	343.8	391.2	1100			99% < EAR
Copper (mg)	18.5	6.9	10.4	13.5	17.6	22.6	27.7	540			99% < EAR
Fluoride (mg)	2.5	7.5	0.1	0.2	0.7	2.1	5.5		2		26% > AI
Iodine (μg)	14.8	8.5	6.4	8.9	12.8	18.5	25.6	73			99% < EAR
Iron ** (mg)	11.0	2.0	8.5	9.6	10.9	12.4	13.7	16.4/16.8			91% < EAR
Magnesium (mg)	173.5	35.3	129.8	148.5	171.2	196.2	219.9	200			78% < EAR
Manganese (mg)	5.4	1.3	3.8	4.4	5.3	6.2	7.0		1.9/1.6		99% > AI
Phosphorus (mg)	937.9	251.6	644.8	757.5	905.9	1084.5	1270.5	1055			72% < EAR
Potassium (mg)	1246.8	440.8	726.3	927.6	1193.5	1510.5	1833.9		4500		2% > AI
Selenium (μg)	47.0	21.3	22.6	31.4	43.8	59.2	75.5	35			32% < EAR
Sodium (mg)	1556.9	397.8	1068.3	1273.2	1526.5	1809.6	2082.6		1200	1600	81% > AI
Zinc ** (mg)	11.7	2.6	8.6	9.9	11.5	13.4	15.2	11.5/10.6			58% < EAR
Biotin (μg)	13.2	7.0	5.9	8.2	11.8	16.6	22.3		20		15% > AI
Folate (μg DFE)	153.4	36.1	109.5	127.6	150.2	175.9	201.2	250			99% < EAR
Pantothenic acid (mg)	1.6	0.3	1.2	1.4	1.6	1.7	1.9		4		0.01% > AI
Vitamin A (μg RAE)	271.3	85.4	169.1	209.7	262.1	323.4	384.9	445/420			96% < EAR
Thiamin (mg)	0.6	0.1	0.5	0.5	0.6	0.7	0.8	0.7			75% < EAR
Vitamin B12 (μg)	1.4	0.6	0.7	1.0	1.4	1.8	2.2	1.5			58% < EAR
Riboflavin (mg)	0.7	0.3	0.4	0.5	0.6	0.8	1.0	0.8			74% < EAR
Niacin (mg)	11.6	4.0	6.9	8.7	11.1	14.0	16.9	9			28% < EAR
Vitamin B6 (mg)	1.0	0.3	0.6	0.8	1.0	1.2	1.4	0.8			30% < EAR
Vitamin C (mg)	31.0	10.1	18.7	23.7	30.0	37.3	44.3	39			79% < EAR
Vitamin D (μg)	1.2	0.6	0.5	0.7	1.1	1.5	2.0	10			99% < EAR
Vitamin E (mg)	4.6	1.1	3.2	3.8	4.5	5.3	6.1	9			99% < EAR
Vitamin K (μg)	51.7	16.5	32.3	39.8	49.8	61.6	73.7		60		28% > AI

* Based on IOM recommendation of 14 g/1000 kcal of fibers and adjusted by the median energy of each age group. ** The recommended nutrient intakes (RNIs) of iron and zinc with low bioavailability from the WHO and FAO. Abbreviations used: Standard Deviation (SD); Estimated Average Requirement (EAR); Adequate Macronutrient Distribution Ranges (AMDR); Adequate Intake (AI); Saturated Fatty Acids (SFA); and Dietary Folate Equivalents (DFE)

**Table 4:** Percentage of micronutrient intakes below the EAR among 4–8 and 9–13-year-old Nigerian children across socio-economic levels.

	Total			4–8-Year-Olds *			9–13-Year-Olds *		
Nutrient	4–8 Years	9–13 Years	*p* Value	Highest	Middle	Lowest	Highest	Middle	Lowest
Calcium	99%	99%	0.9999	99%	99%	99%	99%	99%	99%
Copper	99%	99%	0.9999	99%	99%	99%	99%	99%	99%
Iron	62%	91%	<0.0001	61%	61%	64%	90%	90%	91%
Magnesium	13%	78%	<0.0001	13%	13%	12%	78%	78%	78%
Phosphorus	1%	72%	<0.0001	1%	1%	1%	71%	71%	72%
Zinc	44%	58%	<0.0001	43%	43%	45%	56%	58%	59%
Folate	74%	99%	<0.0001	73%	74%	74%	99%	99%	99%
Vitamin A	67%	96%	<0.0001	66%	67%	67%	96%	96%	95%
Thiamin	28%	75%	<0.0001	28%	28%	28%	75%	76%	75%
Riboflavin	25%	74%	<0.0001	24%	25%	25%	74%	74%	74%
Niacin	8%	28%	<0.0001	8%	8%	8%	28%	27%	28%
Vitamin B6	4%	30%	<0.0001	4%	4%	4%	31%	29%	30%
Vitamin B12	33%	58%	<0.0001	33%	33%	33%	58%	59%	58%
Vitamin C	9%	79%	<0.0001	9%	9%	10%	79%	80%	80%
Vitamin D	99%	99%	0.9999	99%	99%	99%	99%	99%	99%
Vitamin E	97%	99%	0.0001	97%	97%	97%	99%	99%	99%

*p* value: *p* value when comparing percent of children below the EAR between the 4–8 years and 9–13 years age groups. * Comparison of the percent of children below the EAR among three socio-economic levels were conducted within each age group, *p* > 0.05 for all nutrient in the list. Bonferroni correction applied to adjust for multiple comparisons.

## Data Availability

The data presented in this study are available on request from the corresponding author. The datset are not publicly available due to ethical reasons.
